# Dynamics of biochemical attributes and enzymatic activities of pasteurized and bio-preserved tender coconut water during storage

**DOI:** 10.3389/fnut.2022.977655

**Published:** 2022-09-23

**Authors:** R. Pandiselvam, V. Prithviraj, M. R. Manikantan, P. P. Shameena Beegum, S. V. Ramesh, Anjineyulu Kothakota, A. C. Mathew, K. B. Hebbar, Cristina Maria Maerescu, Florin Leontin Criste, Claudia Terezia Socol

**Affiliations:** ^1^Physiology, Biochemistry and Post-Harvest Technology Division, ICAR—Central Plantation Crops Research Institute, Kasaragod, India; ^2^Department of Food Engineering, National Institute of Food Technology Entrepreneurship and Management, Sonipat, Haryana, India; ^3^Agro-Processing and Technology Division, CSIR-National Institute for Interdisciplinary Science and Technology (NIIST), Trivandrum, Kerala, India; ^4^Department of Genetics, University of Oradea, Oradea, Romania

**Keywords:** bio-preservatives, polyphenol oxidase, peroxidase, overall acceptability, enzyme inactivation

## Abstract

The potential of bio-preservatives, namely, nisin, natamycin, and polylysine, as viable alternatives to chemical preservatives for storage of tender coconut water (TCW) during refrigerated storage (5 ± 2°C) was explored. Bio-preservative treatments were carried out after optimized heat treatment (85°C for 5 min) of TCW to establish its storage characteristics. Various concentrations (up to 125 ppm) of bio-preservatives were used for the preservation, and quality parameters of resultant TCW were assessed based on physicochemical characteristics and Food and Agriculture Organization (FAO) guidelines and statistical analysis applied. Analysis of variance (ANOVA) and *post-hoc* test revealed that pH and overall acceptability (OA) are the major governing factors that determine spoilage of TCW (*p* < 0.05). Overall, the polylysine combination was found to be most effective in ensuring quality retention of TCW. It was concluded that pasteurized TCW shelf life could be extended up to 20 days using bio-preservatives.

## Introduction

Coconut (*Cocos nucifera* L.), a member of the *Arecaceae* family, is an important plantation crop in tropical and sub-tropical regions around the globe. The palm has its spherical-shaped drupe called coconut, which consists of an outer husk, i.e., a hard shell, and beneath that the transparent, nutritious, and slightly acidic coconut water. It is commonly known as the coconut tree that supplies a highly nutritious fleshy kernel and the sweet refreshing liquid endosperm referred to as tender coconut water (TCW). The TCW is a natural source of several nutrients and is widely consumed for its numerous therapeutic and health effects (1). TCW is loaded with abundant nutrients, such as sugars, ascorbic acid, and various minerals that include iron, calcium, copper, sodium, potassium, and phosphate (1, 2), making it a highly preferred sports drink (3, 4).

Tender coconut water has been widely used as a curative for different types of ailments, such as heat stroke, kidney disorders, urinary infections, stomach pain, and hot rashes (2). Additionally, it could be used as preventive medicine for sickle cell disease, cholera, and gastroenteritis and is a healthy option for rehydration and diabetes (5, 6). Moreover, various constituents of TCW significantly support the human antioxidant system, and at the same time, it has natural isotonic nature, low calorific value, and good sensorial attributes (6, 7). Nevertheless, this highly nutritious drink undergoes rapid spoilage when exposed to atmospheric conditions due to numerous biochemical reactions (8, 9). Among the deteriorating chemical actions, the enzymatic reaction of peroxidases (PODs) and polyphenol oxidases (PPOs) significantly limits the storage life of TCW. In addition, off-odors, rancid flavors, discolorations, and microbial spoilage further limit its shelf life (2, 10). Hence, bottling and preservation of coconut water immediately after its extraction are indispensable. In this context, various preservation methods, which include different thermal and non-thermal methods, are employed to maintain the keeping quality of TCW (1).

Conventionally, various heat treatments are employed to prevent quality deterioration in coconut water; nonetheless, numerous non-thermal techniques are also utilized. Ozonation (11), filtration (12), ultrasound (13), and high-pressure processing (14) are some of the notable non-thermal methodologies. However, the lack of industrial-scale design and economics of operation are the bottlenecks of these technologies that make them difficult to adopt on a large scale. Additionally, the residues formed in the TCW due to the chemical preservation method greatly limit their application as well, which further paves the way for the use of natural additives. Thus, along with these available industrial technologies, the addition of different types of bio-preservatives is a promising method, which appreciably extends the shelf life of TCW. For instance, the effect of various additives on the quality aspects of TCW analyzed by Mahnot et al. (15) elucidated that different concentrations of ascorbic acid, citric acid, and L-cysteine helped in maintaining the sensorial attributes of coconut water (15). Similarly, the efficiency of 25–75 ppm nisin in preserving the various organoleptic characteristics of micro-filtered coconut water was examined by Sumonsiri (12). It demonstrated that the addition of 50 ppm nisin could be recommended to maintain the sensory properties of TCW during cold storage.

Mahnot et al. (15) and Sumonsiri (16) have conducted studies on the quality deteriorations and the maintenance of the sensory profile of coconut water. The effect of the combination of bio-preservatives on the shelf life of TCW has not been explored yet. Instead of using a single bio-preservative, the combination of different bio-preservatives could reduce the severity of thermal processing. The effect of bio-preservatives, such as natamycin and polylysine, on the shelf life extension of TCW, is not yet explored. To the best of our knowledge, studies on the effects of heat treatment and bio-preservatives on the storage life and/or shelf life of TCW are scarce. Hence, the objective of this study was to examine the effects of bio-preservatives, such as nisin, natamycin, and polylysine, on the storage endurance of pasteurized TCW.

## Materials and methods

### Raw materials

Fresh tender coconuts (var. West Coast Tall [WCT]) of 7-month maturity were harvested from the Farm Section, the Indian Council of Agricultural Research-Central Plantation Crops Research Institute (ICAR-CPCRI), Kasaragod, Kerala, India. The harvested nuts were transported to Agro Processing Complex, and the surface of tender coconuts was cleaned with tap water. Later the liquid endosperm, commonly known as TCW, was extracted using a coconut punching machine developed by ICAR-CPCRI, and the extracted water was pooled and mixed in a stainless-steel container. The extracted TCW was stored under −18°C in a deep freezer until further processing.

### Heat treatment

The TCW stored under deep-freezer conditions was brought back to atmospheric temperature and allowed to thaw and equilibrate with the ambient conditions (29 ± 2°C and 62% relative humidity [RH]) prior to heat treatment. For the heat treatment process, a double wall pasteurizer (Harvest Hi-Tech Equipments Pvt. Ltd, Coimbatore, India) with water as a heating medium was used and allowed to preheat up to 84°C. The equilibrated TCW was transferred to a pasteurizer, and its temperature was monitored using the temperature probe. Based on our previous study, the TCW was treated at 84°C for 5 min (10). The TCW was allowed to cool to reach the ambient conditions (29 ± 2°C and 62% RH) following the heating process.

### Packaging and addition of bio-preservatives

The pasteurized TCW was cooled down and divided into ten equal fractions for the addition of bio-preservative (Table 1). The concentration of the bio-preservatives, such as nisin, polylysine, and natamycin, was fixed according to the requirements of food standards. All the samples following treatment were stored at refrigerated conditions (4 ± 1°C) for shelf life studies.

**Table 1:** Treatmentdetails of different combinations of bio-preservatives and their concentration.

**Batch**	**Nisin**	**Polylysine**	**Natamycin**
**Name**	**(ppm)**	**(ppm)**	**(ppm)**
T-0	-	-	-
T-1	50	-	5
T-2	75	-	5
T-3	100	-	5
T-4	125	-	5
T-5	62.5	62.5	5
T-6	-	50	5
T-7	-	75	5
T-8	-	100	5
T-9	-	125	5

### Determination of quality parameters

The following quality parameters were analyzed after the heat treatment. All the experiments were performed in triplicates, and the mean values were computed.

### pH and total soluble solids (TSS)

pH was determined using Testr35 (Eutech Instruments) handheld pH meter. The pH range of equipment varied from 0 to 14 with 0.1 pH accuracy. The TSS was determined using PAL-BX/RI Pocket Refractometer (PAL-BX/RI; ±0.1% accuracy).

### Turbidity

Turbidity was measured in Nephelometric Turbidity Units (NTU) using Eutech TN-100 Turbidimeter. The equipment was designed to measure turbidity in the range of 0–2,000 NTU with ±1 accuracy.

### Titratable acidity

Titratable acidity was determined following the method suggested by Thimmaiah (16) and Beegum et al. (17). The malic acid was used as standard since the organic acid was found to be the predominant acid in TCW (17, 18). TA was calculated using 0.0098 N NaOH and phenolphthalein indicator. The results are expressed as the mass of Malic acid (equivalent weight = 67.05 g) in 100 ml.

### Total phenolic content

The TPC of the TCW was assessed using Folin-Ciocalteu's method (19) with slight modifications. In brief, gallic acid (1 μg/ml) stock solution was prepared and later made up to 100 ppm by the addition of distilled water. To prepare a standard curve, 10, 20, 30, 40, and 50 ppm concentrations of the stock solution were taken out. In addition, a sample of 0.1 ml was taken from each treatment for analysis with two replications. Later, all the solutions were made up to 1 ml using distilled water, and an extra blank with distilled water was kept to avoid error. All solutions were mixed with 0.2 ml of 50% Folic-Ciocalteu Reagent (FCR) and 2 ml of 7% Na_2_CO_3_, and the mixture was shaken well and kept in the dark for 1 h incubation at room temperature (23 ± 2°C and 62% RH). Spectrophotometric absorbance was recorded using a Shimadzu UV-160 spectrophotometer at 750 nm, and the results were expressed as gallic acid equivalents (GAEs) in mg/100 L.

### PPO activity

Polyphenol oxidase enzymatic activity was determined according to the method described by Porto et al. (20) with slight modifications. In brief, the treated TCW sample of 1 ml was added to 1 ml of potassium phosphate buffer (pH = 6) containing catechol (0.1 M) as reacting substrate. The reaction was carried out at room temperature and absorbance was measured using a spectrophotometer (Shimadzu UV-160) at 425 nm for every 15 s up to 30 min. The enzymatic activity was calculated using equation-1. PPO activity was expressed as units of enzyme activity wherein a unit of enzyme activity is the amount of enzyme that causes a change of 0.001 in the absorbance per minute.


(1) $$\begin{array}{l} PPO\;Activity=\frac{k}{0.001} \end{array}$$

k = slop of the absorbance vs. time plot.

### POD activity

Peroxidase activity was determined according to the method described by Porto et al. (20) with slight modifications. Phosphate-citrate buffer (pH = 5) with 1% pyrogallol substrate was prepared before analysis. A treated TCW sample of 1 ml was added with 950 μ of buffer solution and 50 μl of 3% H_2_O_2_ as substrate. The reaction was carried out at room temperature, and absorbance was measured using a spectrophotometer (Shimadzu UV-160) at 470 nm for every 15 s up to 5 min. The enzymatic activity was calculated using equation-2. POD activity was expressed in the enzymatic activity unit. One enzyme activity unit is the amount of enzyme that causes a change of 0.001 in the absorbance per minute.


(2) $$\begin{array}{l} POD\;Activity=\frac{k}{0.001} \end{array}$$

k = slop of the absorbance vs. time plot.

### Sensory evaluation

A trained panel between the age group of 20–60 was selected for sensory evaluation of treated samples. The procedure adopted from our previous study (10) was followed for sensory evaluation. The panel assigned scores on the 9-point Hedonic Scale based on aroma, freshness, sweetness, burnt, off-flavor, color, and overall acceptability (OA). The OA parameter was used for response surface modeling.

### Statistical analysis

The observations and their variations with storage days were subjected to statistical analysis using Minitab 19 (Minitab, LLC). Each response was represented as separate time series plot to understand the individual variations during the storage period. Also, each response was subjected to a two-way analysis of variance (ANOVA) to understand the effect of different treatments and storage periods.

## Results and discussions

### Effect of pasteurization and bio-preservatives combinations on pH of TCW

**Figure 1:**
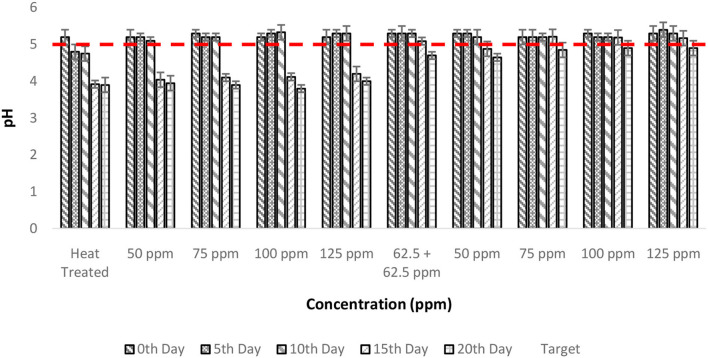
pH vs. concentration plot of bio-preservatives during the storage period.

The initial pH of TCW before treatment was found to be 5.2 ± 0.1, which was maintained even after the heat treatment of TCW. Also, the addition of bio-preservatives did not modify the pH significantly on the 0th day. Previous studies on the effect of pH also found a similar result emphasizing the fact that heat treatment did not significantly alter the pH of TCW (21). The little variation in the pH observed was due to the settlement of solids (21). However, 5 days after the treatment, the pH of heat-treated samples showed a decline and reached values below 5 (indicated by the red dotted line in Figure 1), which is the recommended minimum pH for TCW as per Food and Agriculture Organization (FAO) guidelines (22). The decrease in pH suggests the growth of microorganisms, which produce lactic acid and acetic acid through the consumption of sugar as observed in sugarcane juice and coconut inflorescence sap (23–25). Similar observations were made in apple juices where the low pH indicated the growth of microbes (26).

Treatment with a polylysine combination (75 ppm) could hold the recommended pH up to 15 days of storage, whereas even the highest concentrations (125 ppm) of nisin could only keep up the quality TCW for 10 days. The two-way ANOVA found that both the storage period and treatment had a significant influence on pH levels (*p* < 0.001; Table 2). Furthermore, *post-hoc* multiple comparisons of control and treatments revealed that treatments, T6–T10, are significantly different from the rest, further corroborating the utility of polylysine in maintaining the pH levels of TCW. The reason for this differential behavior might be in the operating pH range of bio-preservatives since polylysine showed bacteriostatic behavior over a pH range of 5–8 (27, 28) whereas nisin was found to be effective when the pH is 4 (29), which in itself was acceptable for TCW.

**Table 2:** Analysis of variance (ANOVA) to understand the statistical effect of storage period and treatments on the quality of tender coconut water.

**Source**	**DF**	**Adj SS**	**Adj MS**	**F-Value**	***P*-Value**
**pH**
Storage Period	4	6.975	1.74382	23.94	0.000
Treatment	9	2.733	0.30371	4.17	0.001
Error	36	2.622	0.07284		
Total	49	12.331			
**TSS**
Storage Period	4	1.870	0.46750	12.65	0.000
Treatment	9	3.005	0.33389	9.04	0.000
Error	36	1.330	0.03694		
Total	49	6.205			
**TA**
Storage period	4	0.000007	0.000002	2.17	0.092
Treatment	9	0.00003 0	0.000003	4.34	0.001
Error	36	0.000028	0.000001		
Total	49	0.000064			
**Turbidity**
Storage period	4	87370	21842.5	31.12	0.000
Treatment	9	47212	5245.7	7.47	0.000
Error	36	25270	701.9		
Total	49	159851			
**PPO**
Storage period	4	0.00022	0.000055	1.62	0.190
Treatment	9	1.46582	0.162869	4839.77	0.000
Error	36	0.00121	0.000034		
Total	49	1.46725			
**POD**
Storage period	4	0.000067	0.000017	0.57	0.688
Treatment	9	0.014532	0.001615	55.09	0.000
Error	36	0.001055	0.000029		
Total	49	0.015654			
**Phenolic content**
Storage Period	4	0.04983	0.012458	5.89	0.001
Treatment	9	0.04429	0.004922	2.33	0.035
Error	36	0.07617	0.002116		
Total	49	0.17029			
**Overall acceptability**
Storage period	4	149.23	37.308	33.60	0.000
Treatment	9	39.71	4.412	3.97	0.001
Error	36	39.97	1.110		
Total	49	228.91			

### Effect of pasteurization and bio-preservatives combination on TSS of TCW

**Figure 2:**
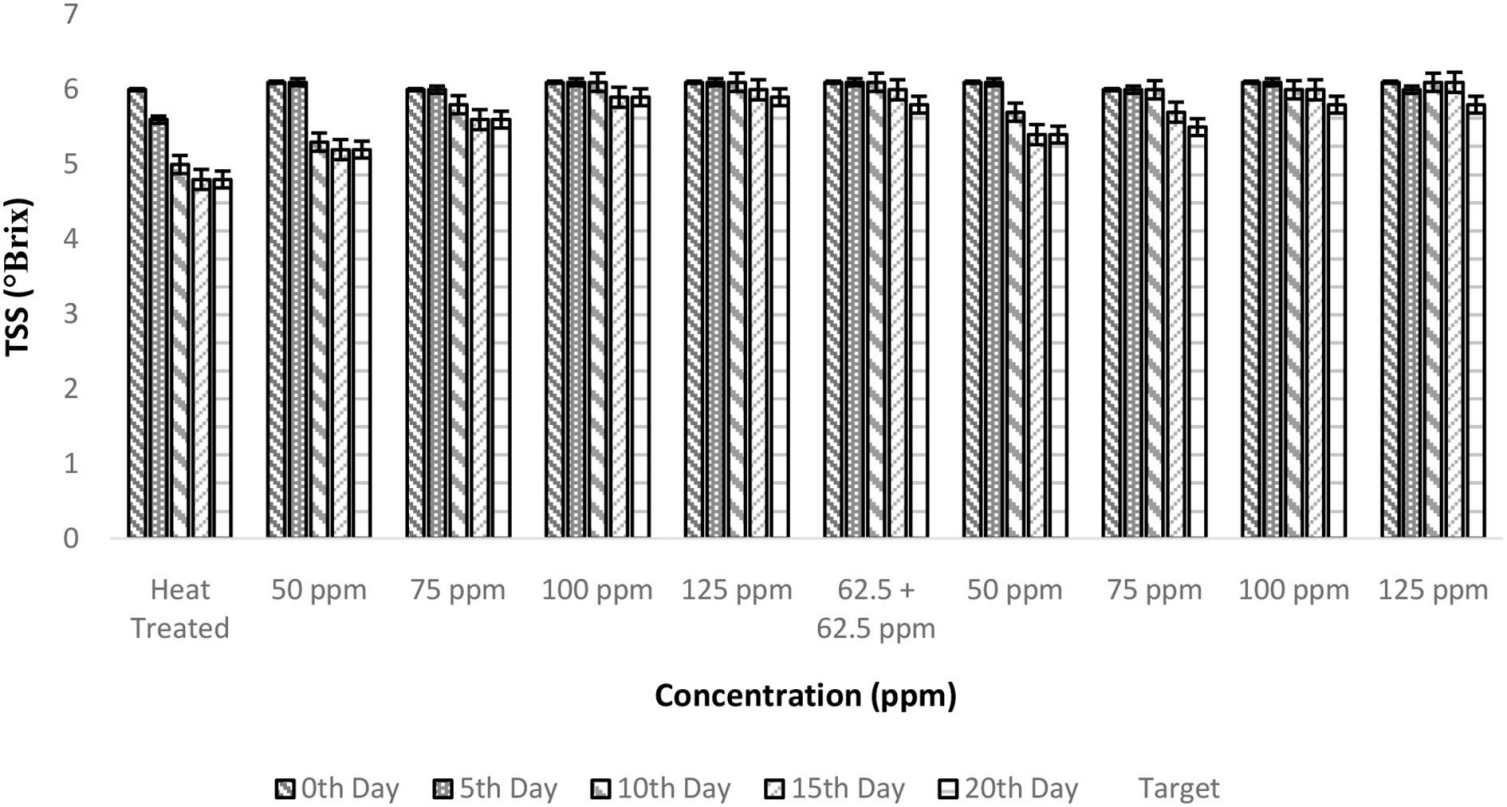
Total soluble solids (TSS) vs. concentration plot of bio-preservatives during the storage period.

The TSS of fresh TCW was found to be 6 ± 0.1° Brix, which remained unaltered even after the addition of bio-preservatives. Overall, a negative correlation was observed between TSS and storage period; however, a stable behavior was found with an increase in treatment concentration (Figure 2). The FAO guidelines recommend a minimum of 5° Brix for bottled TCW, which was maintained by all the treated samples throughout the 20-day period of the experiment. The constant TSS values during storage show the inhibition of microbial activity by bio-preservatives (30, 31), which have innate antimicrobial activities to inhibit the growth of spoilage and pathogenic microbes (32). The mode of action of nisin against microbes is cytoplasmic exudation and cell-wall biosynthesis (33). The addition of polylysine will destroy the cell membranes of the target microorganisms and inhibit the microbial respiratory system, thereby achieving bacteriostatic effects (34, 35). The two-way ANOVA indicated that both storage period and treatment conditions had a significant effect on TSS levels due to a drop in TSS levels from the 0th day (Table 2). Even though the samples of treatments, i.e., T2–T10, maintained TSS levels above 5, there was a significant drop from the initial level (6 ± 0.1 °Brix) for T2 treatment, which was indicated in the *post-hoc* comparison test. Therefore, a higher concentration (>75 ppm) of nisin or combination with polylysine is required to retain the quality of TCW during storage.

### Effect of pasteurization and bio-preservatives combination on TA of TCW

**Figure 3:**
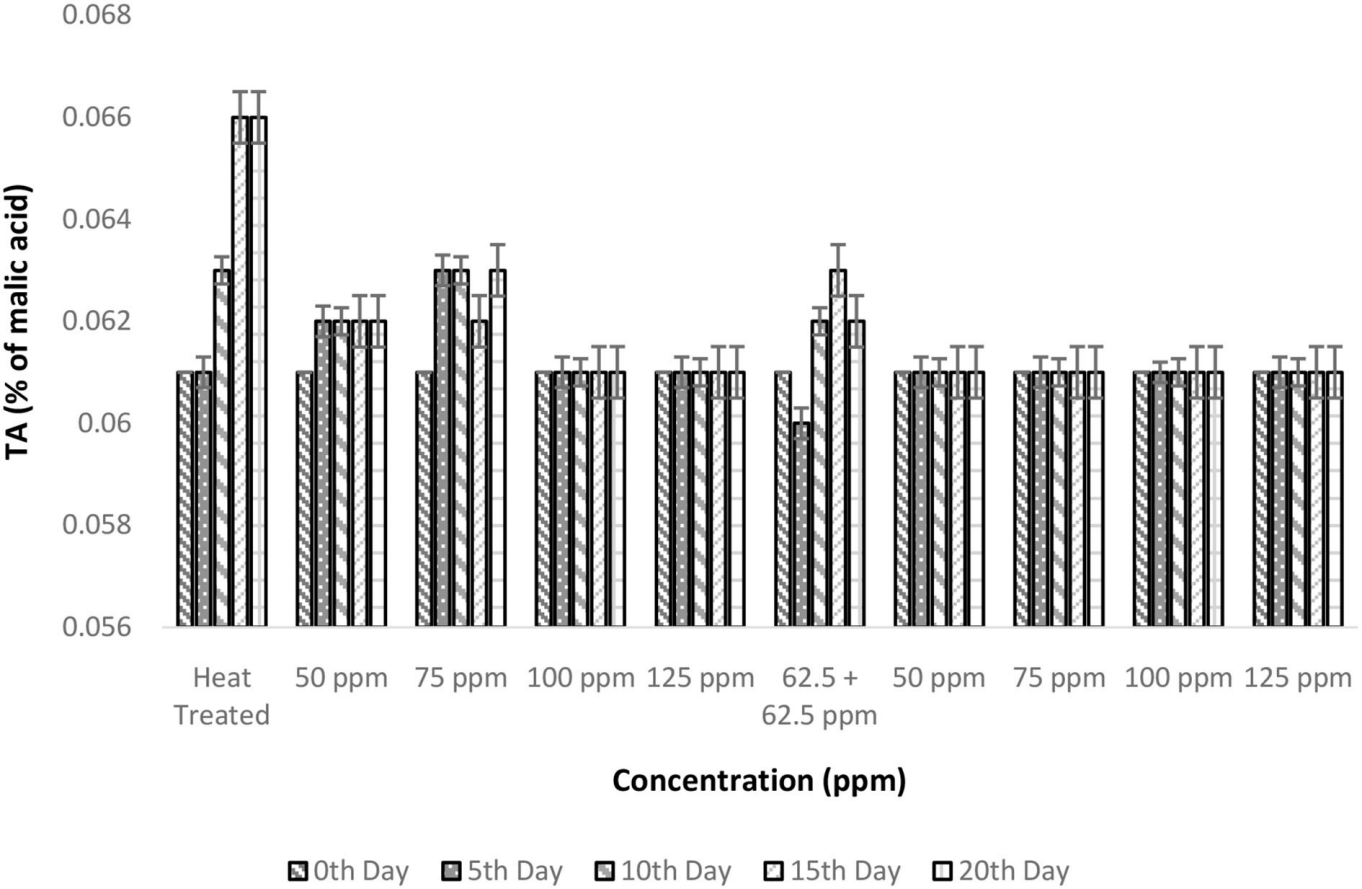
Titratable acidity vs. concentration plot of bio-preservatives during the storage period.

The TA that was assessed using malic acid as standard was found to be 0.061 ± 0% in the fresh TCW. It remained almost constant even after heat treatment and the addition of bio-preservative. A similar result was reporte‘d by Sun et al. (35) for the orange juice treated with thermosonication (input power of 1,000 W and a frequency range of 20–25 kHz) + polylysine. Barring the treatments, i.e., T1, T2, T3, and T6, all others have maintained TA in the range of 0.061 ± 0.005% of malic acid. The increase in TA observed in control after 5 days is due to the process of fermentation in TCW that resulted in the formation of alcoholic compounds (Figure 3). The two-way ANOVA indicated that only treatment conditions have a significant effect on TA (*p* = 0.001); this is due to the static nature of TA during the storage period in the majority of treatments (Table 2). In addition, the treatments, i.e., T1, T2, and T3, are categorized in a group during *post-hoc* Tukey's test attributable to the spoilage caused in these treatments at nisin concentrations less than or equal to 75 ppm.

### Effect of pasteurization and bio-preservatives combination on the turbidity of TCW

**Figure 4:**
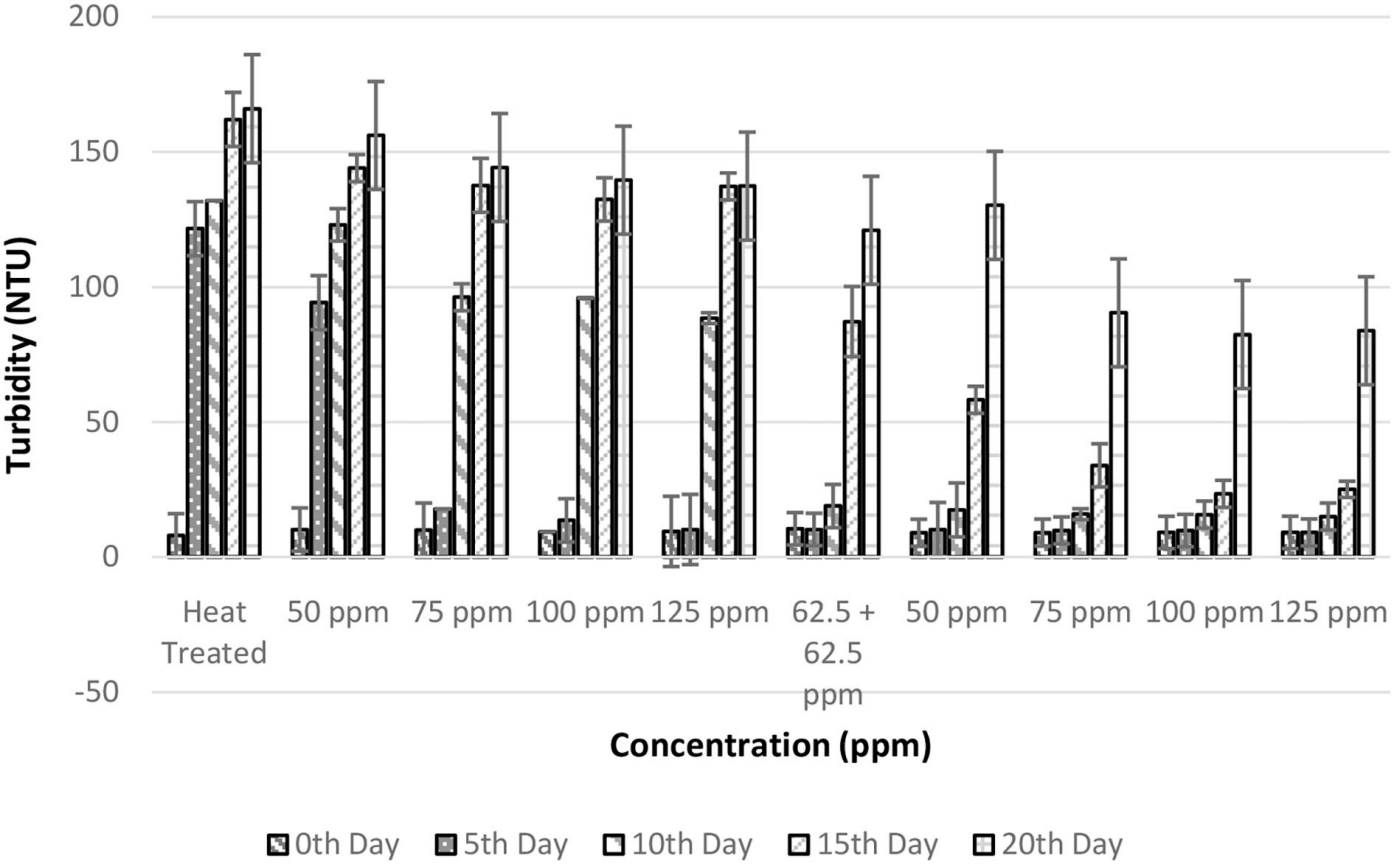
Turbidity vs. concentration plot of bio-preservatives during the storage period.

The turbidity values exhibited the largest numerical variations than all other responses making it the most sensitive response. Initial turbidity values of fresh TCW were found to be 8.05 ± 1 NTU; however, this increased to 9.39 ± 0.704 NTU after the addition of bio-preservatives. The small increase in turbidity may be due to the partial precipitation of suspended insoluble particles in the TCW (36). The turbidity values significantly increased from the 5th day of storage for the treatments, namely, T1 and T2, indicating spoilage of the same (Figure 4). This significant increase in turbidity could be due to the consumption of macromolecular nutrients by microorganisms, which breaks down larger molecules causing an increase in suspended particles indicating microbial growth (30). In addition, the two-way ANOVA indicated that both the storage period and treatments had a significant effect (*p* < 0.05) on turbidity (Table 2). Further analysis of multiple comparisons with Tukey's test indicated that treatments T1–T5 were not statistically different. These samples had a significant increase in turbidity after the 5th day along with a cloudy appearance. From a consumer perspective, TCW is preferred as a clear liquid devoid of any sediment. The polylysine combinations (of concentrations above 75 ppm) held the turbidity below 34 NTU for up to 15 days. However, the treatment with nisin alone could only last up to 5 days without a significant increase in turbidity.

### Combinatorial effect of pasteurization and bio-preservatives on PPO activity in TCW

**Figure 5:**
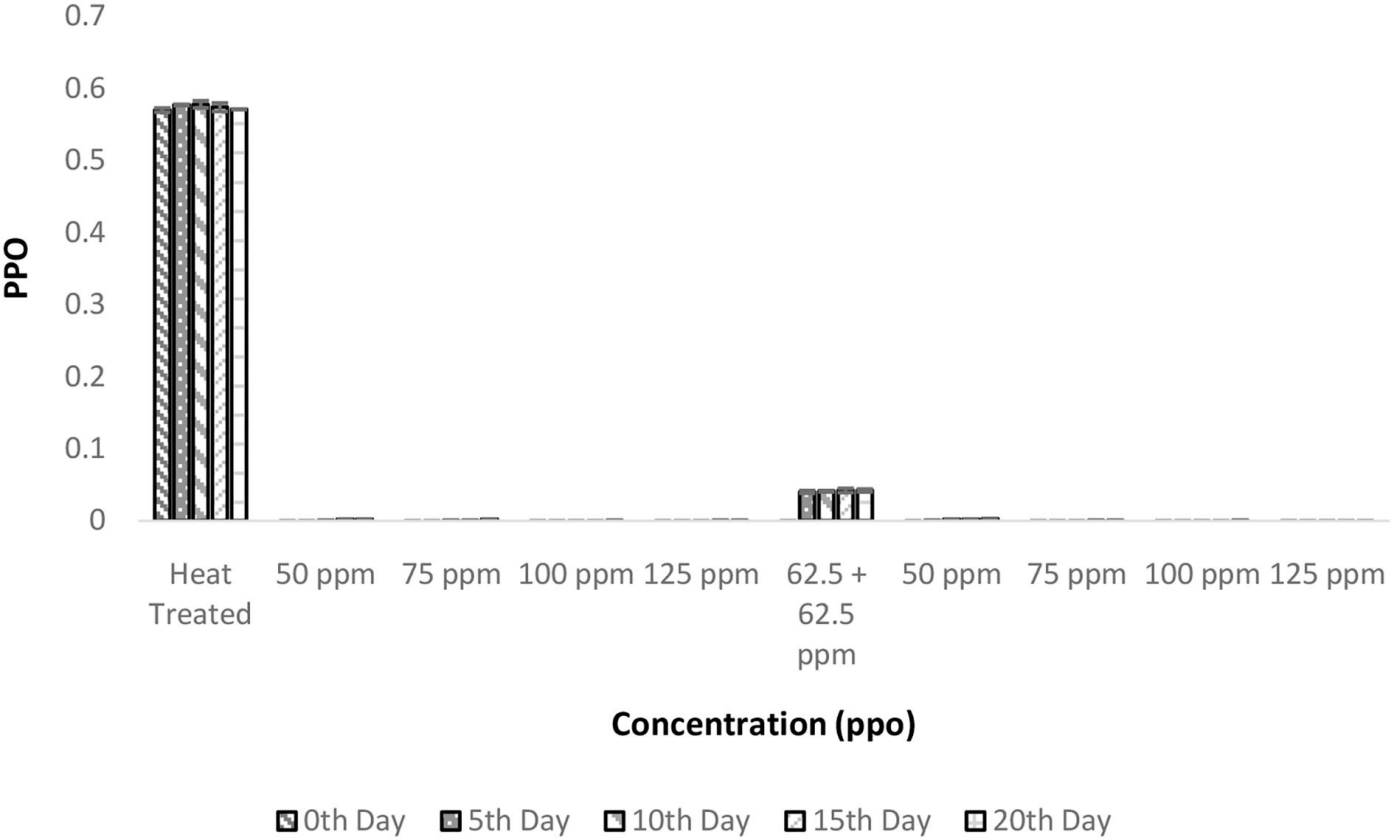
Polyphenol oxidase (PPO) vs. concentration plot of bio-preservatives during the storage period.

The PPO enzyme activity was reduced to negligible units after the heat treatment and this negligible activity remained constant even after 20 days of storage (Figure 5). It is interesting to observe that there was a considerable decrease in PPO activity after the addition of bio-preservatives similar to the observation in the shelf life study of palm sap, which could be due to a reduction in pH following the addition of nisin (37). Similar reductions in PPO activity with the addition of polylysine were observed in lettuce (38). The addition of nisin influences the pH of solutions (37) and, thus, change in the pH of the solution from the optimal pH required for PPO activity (pH = 7) might have decreased the activity of PPO. Similar observations of a decrease in PPO activity were made upon the addition of polylysine to fresh-cut lettuce (38). The PPO values remained within range throughout the storage period for all the treatments. Also, ANOVA deduced that there was a significant influence of various treatments on PPO activity (*p* < 0.05). The *post-hoc* test confirms that all the bio-preservative-added treatments had significant differences from the control sample that was only heat treated. Although all the treatments were found to perform well and the lowest concentrations of bio-preservatives (50 ppm nisin or 50 ppm polylysine) are preferred.

### Combinatorial effect of pasteurization and bio-preservatives on POD activity in TCW

**Figure 6:**
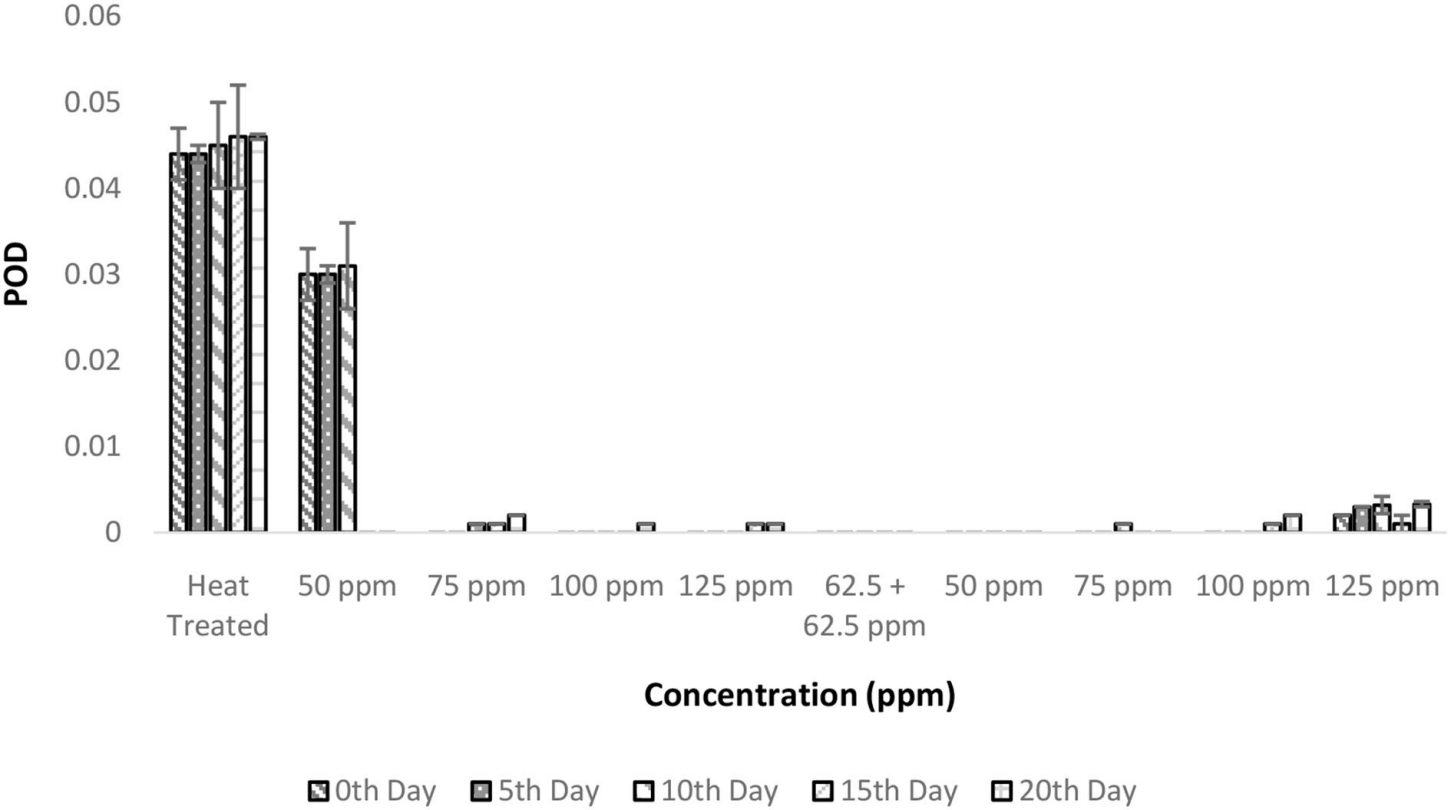
Peroxidase activity (POD) vs. concentration plot of bio-preservatives during the storage period.

The POD activities showed a similar trend as that of PPO in terms of total activity. Heat treatment conditions were sufficient enough to inactivate the enzymatic activity throughout the experimental period (Figure 6). The inactivation of POD follows a complex process in which the denaturation of the enzyme is due to various forces, such as hydrogen bonding, hydrophobic interaction, and electrostatic forces, which increase with temperature (39). The addition of bio preservatives has reduced the enzymatic activity to untraceable levels so that throughout the storage period, the TCW could retain its quality (Figure 6). The ANOVA suggested that various treatment conditions exhibited significant effects on POD activity and the storage period, which was clearly visible in the T2 treatment. It can also be interpreted as the behavior of TCW in good enzymatic stability after heat treatment, which was further enhanced by bio-preservatives. However, other dependent variables are causing the spoilage of products despite the suppressed enzymatic activity. The *post-hoc* comparison reveals that all treatments were different from the control treatment T1. Although all the treatments were found to perform adequately, the lowest concentrations (either 50 ppm nisin or 50 ppm polylysine) of preservatives are preferred.

### Effect of pasteurization and bio-preservatives combination on phenolic content of TCW

**Figure 7:**
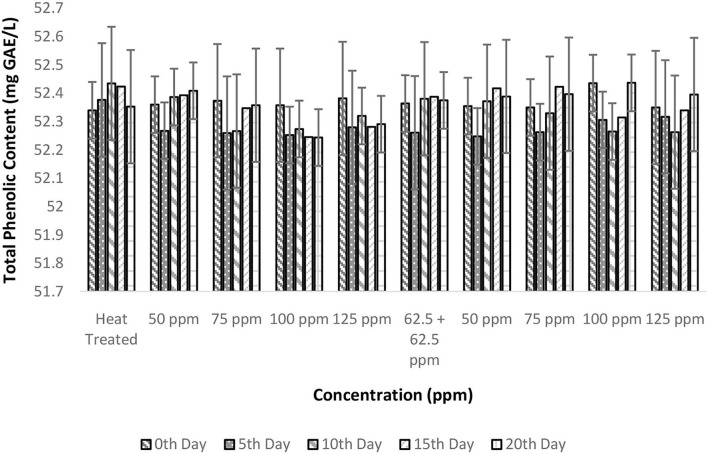
Total phenolic content vs. concentration plot of bio-preservatives during the storage period.

The phenolic content of various treatments exhibited a mean value of 52.34 (mg GAE/L). Although variations in the TPC of different treatments were observed, a definite trend could not be deduced (Figure 7). There was a significant difference in TPC with respect to treatment conditions and storage period (*p* < 0.05) based on ANOVA (Table 2). However, the R^2^ values of the general linear model were below 50% indicating the spread of data points and a lack of trend. In addition, the *post-hoc* test reveals that various treatments were not significantly different; hence, phenolic content of TCW was affected by neither bio-preservative treatments nor storage period. Similar results of non-significant variations of phenolic content were observed during the storage study of carrot juice preserved with nisin (40).

### Effect of pasteurization and bio-preservatives combination on OA of TCW

**Figure 8:**
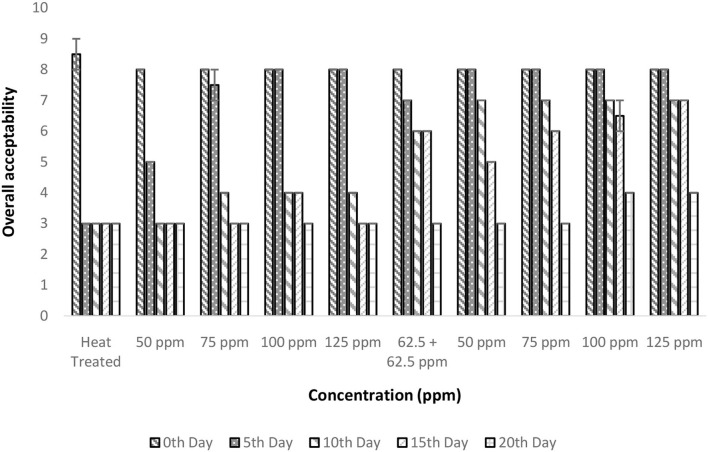
Overall acceptability vs. concentration plot of bio-preservatives during the storage period.

The OA was considered the prime factor as it underlies consumer acceptance. There is a significant influence on OA by treatment conditions and storage period (*p* < 0.05; Figure 8). Only the polylysine treatment (125 ppm) could retain OA for up to 15 days. The heat treatment even though was found to address the fundamental problem of enzymatic reaction; it was failed to maintain the desirable properties of TCW during storage. This was evident from the poor OA score of 3 after 5 days of treatment in control (T1). A similar quality loss was experienced immediately after heat treatment for TCW in overall quality and pleasantness (41). In addition, the *post-hoc* analysis suggested that treatments with polylysine were having significant differences from the control treatment. Also, the results indicated that the polylysine combinations are more effective than nisin treatments. The general linear model developed was having satisfactory R^2^ (>80%) values indicating a proper governing model that correlates treatment condition and storage period with that of overall acceptance.

## Conclusion

The optimized heat treatment for TCW was adequate to suppress the enzymatic activity of both PPO and POD to acceptable levels even after 20 days of storage. However, the biochemical and sensory properties of TCW were found to degrade within 5 days of storage hence bio-preservative treatment was found essential for long-term storage. TCW samples treated with 4 varying concentrations of nisin (50, 75, 100, and 125 ppm) exhibited spoilage on the 10th day after treatment due to a significant drop in pH and OA attributed to the fermentation reaction. The treatments with polylysine (125 ppm) were found to hold TCW quality up to 15 days. In addition, the minimal level of polylysine of 50 ppm maintains TCW quality until the 10th day, which then underwent a drop in pH below the FAO-recommended quality features for TCW. Hence treatment of 75 ppm polylysine or 62.5 ppm combination of nisin and polylysine was required to maintain satisfactory TCW quality till the 20th day. The ANOVA indicated that storage period and treatment concentration are directly linked with pH levels. Bottling and preservation of TCW have huge research and commercial potential, and this investigation provides a way forward for the use of bio-preservatives in preserving TCW.

## Data availability statement

The original contributions presented in the study are included in the article/supplementary material, further inquiries can be directed to the corresponding authors.

## Author contributions

VP: investigation, data curation, methodology, and writing original draft. RP: resources, conceptualization, methodology, and writing original draft. MM: conceptualization, methodology, supervision, writing review, and editing. PB: methodology and writing original draft. SR: methodology, writing review, and editing. AK: methodology, software, and data curation. AM and KH: supervision, writing review, and editing. CM: resources, statistical analysis, and funding. FL: resources, review, and editing. CS: data curation and software. All authors contributed to the article and approved the submitted version.

## Funding

This study was funded by ICAR-All India Coordinated Research Project on Post-Harvest Engineering and Technology (AICRP on PHET), Ludhiana, India (project number: 1000767025).

## Conflict of interest

The authors declare that the research was conducted in the absence of any commercial or financial relationships that could be construed as a potential conflict of interest.

## Publisher's note

All claims expressed in this article are solely those of the authors and do not necessarily represent those of their affiliated organizations, or those of the publisher, the editors and the reviewers. Any product that may be evaluated in this article, or claim that may be made by its manufacturer, is not guaranteed or endorsed by the publisher.
